# Display of VP1 on the Surface of Baculovirus and Its Immunogenicity against Heterologous Human Enterovirus 71 Strains in Mice

**DOI:** 10.1371/journal.pone.0021757

**Published:** 2011-07-01

**Authors:** Tao Meng, Annasaheb B. Kolpe, Tanja K. Kiener, Vincent T. K. Chow, Jimmy Kwang

**Affiliations:** 1 Animal Health Biotechnology, Temasek Life Sciences Laboratory, Republic of Singapore; 2 Department of Microbiology, Yong Loo Lin School of Medicine, National University of Singapore, Republic of Singapore; Duke University School of Medicine, United States of America

## Abstract

**Background:**

Human Enterovirus 71 (EV71) is a common cause of hand, foot and mouth disease (HFMD) in young children. It is often associated with severe neurological diseases and has caused high mortalities in recent outbreaks across the Asia Pacific region. Currently, there is no effective vaccine and antiviral agents available against EV71 infections. VP1 is one of the major immunogenic capsid protein of EV71 and plays a crucial role in viral infection. Antibodies against VP1 are important for virus neutralization.

**Methodology/Principal Finding:**

In the present study, infectious EV71 viruses were generated from their synthetic complementary DNA using the human RNA polymerase I reverse genetics system. Secondly, the major immunogenic capsid protein (VP1) of EV71-Fuyang (subgenogroup C4) was displayed on the surface of recombinant baculovirus Bac-Pie1-gp64-VP1 as gp64 fusion protein under a novel White Spot Syndrome Virus (WSSV) immediate early ie1 promoter. Baculovirus expressed VP1 was able to maintain its structural and antigenic conformity as indicated by immunofluorescence assay and western blot analysis. Interestingly, our results with confocal microscopy revealed that VP1 was able to localize on the plasma membrane of insect cells infected with recombinant baculovirus. In addition, we demonstrated with transmission electron microscopy that baculovirus successfully acquired VP1 from the insect cell membrane via the budding process. After two immunizations in mice, Bac-Pie1-gp64-VP1 elicited neutralization antibody titer of 1∶64 against EV71 (subgenogroup C4) in an *in vitro* neutralization assay. Furthermore, the antisera showed high cross-neutralization activities against all 11 subgenogroup EV71 strains.

**Conclusion:**

Our results illustrated that Bac-Pie1-gp64-VP1 retained native epitopes of VP1 and acted as an effective EV71 vaccine candidate which would enable rapid production without any biosafety concerns.

## Introduction

Human enterovirus 71 (EV71) is a common cause of hand-foot-and mouth disease (HFMD) in young children below 6 years old [1]–[6]. EV71 infections usually are mild, but occasionally lead to severe diseases such as aseptic meningitis, poliomyelitis-like paralysis, and possibly fatal encephalitis [7]. Outbreaks of EV71 with significant morbidity and mortality have been reported worldwide and especially in the Asia-Pacific region. EV71 is associated with fatal cases of HFMD during the large outbreaks in Malaysia in 1997 [2], Taiwan in 1998, 2000 and 2001 [3]–[4], Australia in 1999 [6], Singapore in 2000 [6], [8] and China (2008). From 1999 to 2010, HFMD outbreaks caused by EV71 have affected more than 500,000 children and resulted in more than 200 deaths in China. In fact, after the eradication of poliovirus, EV71 is now regarded as the most important neurotropic enterovirus and a threat to global public health [8]–[11].

Like poliovirus, EV71 is a small, nonenveloped, positive-stranded RNA viral pathogen within the *Picornaviridae* family. The genome of EV71 contains a single large coding region flanked by 5′- and 3′- untranslated regions (5′- and 3′- UTR). The coding region is translated to a single polypeptide, which is then processed by viral proteases to yield nonstructural proteins and 4 capsid proteins: VP1, VP2, VP3 and VP4 assembled as pentameric subunits [12]. The VP1 protein is exposed on the surface of virion and usually targeted by host neutralizing antibodies. Therefore, the VP1 gene is thought to play an important role in viral pathogenesis and virulence [13]. Based on the VP1 gene sequence, EV71 is divided into three major genogroups (denoted A, B and C), and various subgenogroups within genogroups B (B1 to B5) and C (C1 to C5) [14].

Currently, there is no commercial antiviral therapy or vaccine against EV71 infection. The prevention and control of EV71 has simply relied on public health surveillance and quarantine. Because VP1 is involved in the recognition of EV71 receptors on the surface of host cells and displays major antigenicity [15]–[17], the usefulness of VP1 to generate a subunit vaccine has recently been highlighted. Several vaccine candidates based on VP1, including synthetic peptides containing VP1 linear neutralizing epitope [20], [21], recombinant VP1 produced in milk samples of transgenic mice [17] or E. coli [17], [18], bacterially or virally expressed VP1 [15], [19] and VP1 DNA vaccine [17], [22], are immunogenic and show good immune response in vaccinated mice. However, over expression of these proteins has been associated with poor solubility, improper folding and the need of expensive purification methods.

Baculovirus, *Autographa californica* nuclear polyhedrosis virus (AcNPV), was initially used as a biological pesticide, but is nowadays best known for its value as a eukaryotic expression vector and a potential vaccine delivery platform [23]. The baculovirus surface display method is based on the expression of foreign proteins fused to the baculovirus gp64 protein [24]. Gp64 is a 64 kDa envelope glycoprotein which is incorporated into viral particles as they bud from the surface of infected cells and it probably mediates penetration of viruses into host cells by adsorptive endocytosis. Gp64 comprises an N-terminal signal peptide (SP) and a mature domain (MD) that includes the transmembrane domain (TM) and cytoplasmic domain (CTD) [25], [26]. The gp64 fusion protein consists of a foreign peptide inserted between SP and mature domain. The fusion protein is translocated to the plasma membrane after expression and incorporated into the baculovirus envelope along with native gp64. This method has been utilized to develop recombinant baculoviruses as a potential vaccine delivery platform [27]–[29]. Foreign proteins that are expressed in the baculovirus system usually are under the control of the conventional baculovirus polyhedrin promoter which acts strongly in Sf9 cell but is less active in mammalian cells. A novel White Spot Syndrome Virus (WSSV) immediate early ie1 promoter was found to be active in both Sf9 cell and mammalian cells, such as Vero, BHK-21, 293T and human colorectal carcinoma (HCT 116) cells [30], [31]. Therefore, the ie1 promoter allows the expression of foreign proteins not only in Sf9 cell but also mammalian cells after transduction of the baculoviral genome into the host.

In this study, we generated an infectious clone of the C4 strain EV71-Fuyang, which led to an EV71 outbreak in Fuyang city of China in 2008 [32]. Virus was produced from its synthetic genomic complementary DNA (cDNA) using the human RNA polymerase I reverse genetics system [33]–[36]. We also successfully generated chimeric EV71 strains with the EV71-Fuyang backbone and VP1 genes from other EV71 subgenogroups. Since the VP1 protein is the most important immunogen eliciting anti-EV71 antibodies and because the recombinant baculovirus is a promising vaccine vehicle, we constructed two recombinant baculoviruses Bac-Piel-gp61-VP1 and Bac-Pph-gp64-VP1. Both expressed the EV71-VP1 (of Fuyang strain) fused with the mature domain of baculovirus gp64 protein under WSSV ie1 promoter (Pie1) and conventional baculovirus polyhedrin promoter (Pph), respectively. We demonstrated that the recombinant baculoviruses successfully displayed VP1 antigen of EV71 on their viral surface and that they induced humoral immune responses against EV71 infection upon *in vivo* immunization in mice.

## Materials and Methods

### Generation of EV71 virus from its synthetic complementary DNA

**Figure 1:**
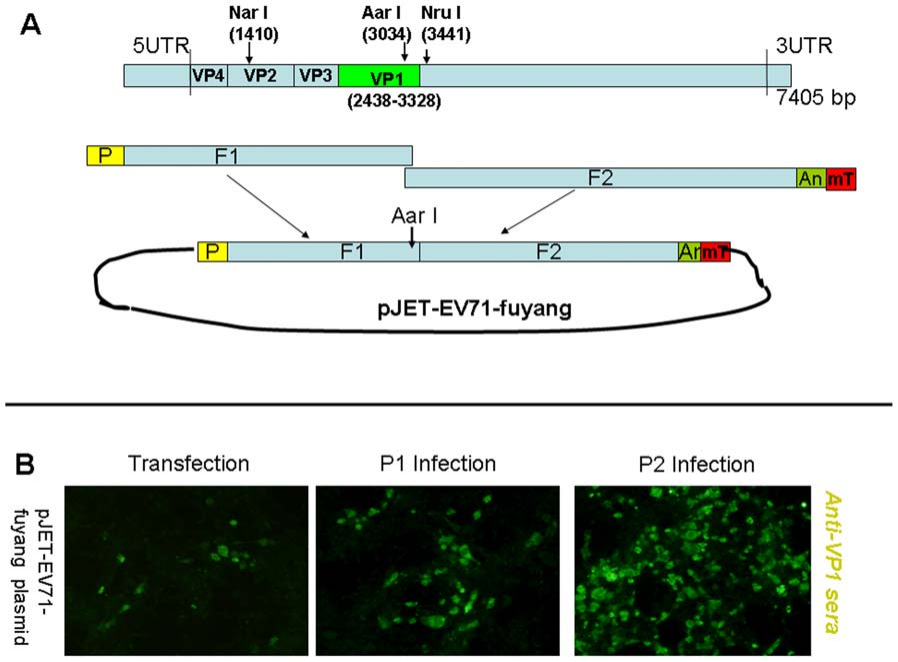
Generation of infectious EV71 from synthetic cDNA by the human RNA polymerase I reverse genetics system. (A) Illustration of cDNA of EV71-Fuyang (EU703813). P: Human RNA I polymerase promoter (225 bp); An: ploy-A tail with 25 adenosines; mT: murine terminator (33 bp). (B) RD cells were directly transfected by plasmid pJET-EV71-fuyang and the VP1 antigen was detected by guinea pig anti-VP1 sera 3 days post-transfection. The recovered EV71 was infectious when passged in RD cells (P1 =  Passage 1; P2 =  Passage 2).

EV71 strain Fuyang/02/08 (EU703813, subgenogroup C4) was generated from its synthetic genomic cDNA using the human RNA polymerase I system. Briefly, the whole cDNA of the virus (7405 bp) with a poly(A) tail containing 25 adenosines was chemically synthesized (Genscript, USA) as two fragments (F1 and F2, Figure 1) with an overlapping region at the internal Aar I restriction enzyme site. Human RNA polymerase I (hRNA pol1) promoter (225 bp) was amplified using primers hRNA-pol1-f and hRNA-pol1-r from SapI/pol vector which is widely used to rescue influenza virus in the reverse genetic system [34]. The F1 was amplified with primers F1-f and F1-r using PfuUltra high-fidelity DNA polymerase (Stratagene), and then joined together with human RNA polymerase I promoter by overlapping PCR, resulting in the product hRNA-pol1-F1. Meanwhile, F2 was cloned with primers F2-f and F2-r containing a murine terminator sequence and named F2-mTer. The hRNA-pol1-F1 and F2-mTer were digested by Aar I (Fermentas), and ligated with pJET1.2/blunt cloning vector (Fermentas) in a triple ligation reaction. The positive recombinant plasmid was cloned in XL1-blue *E. coli* and named pJET-EV71-Fuyang.

For chimeric EV71 viruses, VP1 genes from subgenogroups B1, B3 and C3 (Table 1) flanked with EV71-Fuyang gene fragments (2418-2437 bp at 5′ end and 3329-3450 bp at 3′ end) were synthesized (Genscript) and amplified with primers EV71-f(2418) and EV71-r(3450). Each of these VP1 genes was then joined with the EV71-Fuyang gene fragment (1400-2438 bp, amplified using primers EV71-f(1400) and EV71-r(2438)) by overlapping PCR using primers EV71-f(1400) and EV71-r(3450). After digestion with *Nar*I and *Nru*I (NEB), the PCR products containing heterologous VP1 genes were inserted into pJET-EV71-Fuyang replacing the original VP1 gene of pJET-EV71-Fuyang. These chimeric EV71 constructs were named pJET-EV71-Fuyang (VP1-B1), pJET-EV71-Fuyang(VP1-B3), and pJET-EV71-Fuyang(VP1-C3) and sequences were confirmed by DNA sequencing.

**Table 1:** Primers used for construction of recombinant plasmids.

Primer	Sequence (5′→3′)
hRNA-pol1-f	5′-GCCCCTGCGTGTGGCACG-3′
hRNA-pol1-r	GGTGCAACCCACAGGCTGTTTTAAAATAACCCGGCGGCCCAAAATGC
F1-f	TTAAAACAGCCTGTGGGTTGC
F1-r	GTAAGCACTCGCAGGTGA CATGAATG
F2-f	CATTCATGTCACCTGCGAGTGCTTAC
F2-r	GCCCGGAGTACTGGTCGACCTCCGAAGTTGGGGGGGTTTTTTTTTTTTTTTTTTTTTTTTTGCTATTCTGG
EV71-f(2417)	CTGCAGACGGGCATCATCCAG
EV71-r(3450)	AGTAAGTCGCGAGAGCTGTC
EV71-f(1400)	ACTCAACCCGGCGCCGATG
EV71-r(2437)	CTGGATGATGCCCGTCTGCAG
gp64-MD-f	GAGCACTGCAACGCGCAAATG
gp64-MD-r	GGGTAAGCTTTTAATATTGTCTATTACGGTTTCTAATCATAC
VP1-f	GCATTCTGCCTTTGCGGATCTGCAGGGAGATAGGGTGGCAGATG
VP1-r	CATTTGCGCGTTGCAGTGCTCAAGAGTGGTGATCGCTGTG
gp64-SP-f	TCTCGGTCCGACCATGGTAAGCGCTATTGTTTTATATGTGCTTTTGGCGGCGGCGGCGCATTCTGCCTTTGCGGAT
WSSVie1-f	CCTACGTATCAATTTTATGTGGCTAATGGAGA
WSSVie1-r	CGCGTCGACCTTGAGTGGAGAGAGAGCTAGTTATAA

The purified pJET-EV71-Fuyang and 3 chimeric EV71 constructs were transfected into human rhabdomyosarcoma (RD) cells with Lipofectamine 2000 (Invitrogen) according to the manufacturer's protocol. The transfected cells were lysed after 4 days by 3 freeze-thaw cycles and the supernatant containing viruses was collected for further infection. After 2 passages in RD cells, the generated viruses were harvested and detected with guinea pig anti-VP1 sera (prepared by immunizing with purified His-tagged VP1 expressed in *E. coli*) in IFA (described below).

EV71-Fuyang was used as homologous strain and its VP1 gene was used to construct the recombinant baculoviruses. Eight other wild type EV71 strains (Table 1) kindly provided by Professor Vincent T. K. Chow (National University of Singapore) and 3 chimeric EV71 strains, i.e. EV71-Fuyang(VP1-B1), EV71-Fuyang(VP1-B3), and EV71-Fuyang(VP1-C3) were used as heterologous strains in the neutralization assay. All EV71 viruses were grown in RD cells in Dulbecco modified Eagle medium (DMEM) (Gibco, USA) supplemented with 10% fetal bovine serum (FBS), 6 g/l sodium bicarbonate and antibiotics at 37°C with 5% carbon dioxide and humidity.

### Purification and inactivation of EV71 virus

The RD cells were infected by the reverse genetically engineered EV71 virus (EU703813) at a multiplicity of infection (MOI) of 0.1. Once the cells showed cytopathic effect (CPE), they were harvested and completely lysed by 3 freeze/thaw cycles. Cellular debris was removed by centrifugation at 5,000 x g for 30 min. The virus was purified by precipitation with 7% polyethylene glycol 8000 (PEG 8000) and centrifugation through 30% sucrose cushion at 30,000 g for 4 h. The 50% tissue culture infective dose (TCID_50_) was determined in RD cells using the Reed and Muench formula [37]. The purified virus was heat-inactivated at 56 for 30 min, and the amount of virion protein was quantified by the Bradford assay (Bio-Rad Laboratories, USA).

### Construction of recombinant Bacmid DNA for baculovirus surface display of VP1

The gp64 mature domain (MD) was amplified from wild-type bacmid DNA using primers gp64-MD-f and gp64-MD-r. The VP1 gene was amplified from genomic RNA of EV71 by RT-PCR using primers VP1-f and VP1-r. The gp64-VP1 gene was amplified from an equal molar mixture of purified gp64 MD gene with purified VP1 gene by overlapping PCR using primers gp64-SP-f and gp64-MD-R. The PCR product gp64-VP1 was purified and digested by *Rsr*II and *Hind*III, then inserted into pFast HT A vector. The positive plasmids were verified by DNA sequencing and named pFast-Pph-gp64-VP1. In addition, the baculovirus polyhedrin promoter of pFast-Pph-gp64-VP1 was removed by *Acc*I and *Rsr*II enzymes and replaced by the ie1 promoter of White Spot Syndrome Virus (WSSV), and the new plasmid was named pFast-Pie1-gp64-VP1. The ie1 promoter was amplified from WSSV genomic DNA using primers WSSVie1-f and WSSVie1-r as previously described [31].

Competent DH10Bac *E. coli* cells were then transformed with the recombinant plasmids pFast-Pph-gp64-VP1 and pFast-Pie1-gp64-VP1. After selecting colonies through two rounds of blue/white selection and PCR with M13 forward and reverse primers, recombinant bacmid DNA was isolated from white colonies according to the standard procedure (Bac-to-Bac system, Invitrogen).

### Generation and purification of recombinant baculoviruses

Baculovirus was propagated in *Spodoptera frugiperda* Sf9 III cell lines (ATCC) which were grown at 27°C in serum-free medium SF-900 III (Invitrogen). Procedures for the generation of recombinant baculovirus were carried out according to the manufacturer's manual (Invitrogen). Briefly, 10^6^ Sf9-III cells were cultured in 6-well plates for 1 h. After attachment, 4 µg of recombinant bacmid DNA Bac-Pph-gp64-VP1 or Bac-Pie1-gp64-VP1 was mixed with 10 µl Cellfectin II (Invitrogen) in Grace's insect medium, and then transfected into the Sf9 III cells. Transfected cells were incubated for 5 h at 27°C and the transfection medium was then replaced with fresh medium SF-900 III. After incubation for 72 h at 27°C, the supernatant containing recombinant viruses was used for infecting more fresh Sf9 cells. For large scale viral production, Sf9-III cells were infected at 0.2 MOI in suspension cultures of 2×10^6^ cells/ml. Four days post infection, the supernatant was collected and the recombinant viruses were purified by two rounds of sucrose gradient ultracentrifugation following standard protocols [38]. The purified recombinant baculoviruses were resuspended in PBS and titered by plaque assay.

### Sodium dodecyl sulfate-polyacrylamide gel electrophoresis (SDS-PAGE) and Western blot analysis

The purified baculoviruses and heat-inactivated EV71 were subjected to 8% SDS-PAGE and transferred to nitrocellulose membranes. Two primary antibodies, mouse anti-gp64 monoclonal antibody (1∶10,000, Merk) and guinea pig anti-VP1 serum (1∶2000), were used to detect gp64 fusion proteins in Western blots. The secondary antibodies were horseradish peroxidase (HRP) conjugated goat anti-mouse IgG antibodies (1∶3000, DAKO) or HRP conjugated rabbit anti-guinea pig IgG antobodies (1∶3000, DAKO). The protein bands were visualized by the Amersham ECL Plus Western Blotting Detection Reagents (GE Healthcare).

### Immunofluorescence assay and confocal microscopy to detect expression of recombinant VP1 protein in insect cells

The Sf-9 cells were cultured on glass bottom microwell dishes (MatTek Corporation) to 50% confluency and infected with an MOI of 0.2. Three days after infection, the cells were fixed by methanol/acetone (1∶1) for 10 min at −20°C, rinsed with PBS-T, and blocked with 1% bovine serum albumin for 30 min at 37°C. The cells were incubated with the primary anti-VP1 sera (1∶2000) for 1 h at room temperature, followed by three PBS-T washes. The cells were then incubated with the secondary fluorescein isothiocyanate (FITC)-conjugated goat anti-guinea pig IgG monoclonal antibody (1∶100) for 1 h at room temperature, followed by DAPI (2, 4-diamidino-2-phenylindole) staining at a concentration of 1 µg/ml for 15 min at room temperature and three PBS-T washes. The negative control cells were treated similarly. The FITC signal was detected with an inverted fluorescence microscope (Olympus), and the images were captured by a digital imaging system (Nikon). The cellular localization of recombinant VP1 proteins in Sf9 cells was visualized using a confocal microscope (LSM 510, Zeiss, USA).

### Immunogold Electron Microscopy

Carbon-coated grids were floated on purified baculovirus solution for 30 min, blocked, washed with PBS three times for 5 min, and then exposed to anti-VP1 sera (1∶1000) for 30 min. After two PBS washes, the grids were exposed to anti-guinea pig IgG conjugated with 10-nm gold particles (1∶50) (Sigma) for 30 min. After three more PBS washes, the grids were stained with 2% phosphotungstic acid (Sigma) and examined under the transmission electron microscope JEM-1230 (Jeol).

### Mice Immunization

Five groups of mice, each including six 4-week old female BALB/c mice, were immunized subcutaneously twice on day 0 with 100 µl solution containing 20 µg inactivated EV71, 10^8^ pfu purified Bac-Pph-gp64-VP1, 10^8^ pfu purified Bac-Pie1-gp64-VP1, 10^8^ pfu purified wild-type baculovirus Bac-wt, or RD cell lysate, respectively, with Freund complete adjuvant (Sigma). On day 21, the mice were boosted with the same antigens with Freund incomplete adjuvant (Sigma). Inactivated EV71 was used as positive control, while wild type baculovirus Bac-wt and RD cell lysate served as negative control. 200 µl of blood samples were taken from every mouse by facial bleeding on days 14, 28 and 42, and final bleeding was done on day 56. The sera were harvested for the ELISA and the neutralization titer test.

All animal experiments were carried out in accordance with the Guides for Animal Experiments of the National Institute of Infectious Diseases (NIID), and experimental protocols were reviewed and approved by Institutional Animal Care and Use Committee of the Temasek Life Sciences Laboratory (Project Approval no. TLL-11-002), National University of Singapore, Singapore.

### ELISA for detection of anti-EV71 antibodies

The levels of specific IgG against EV71 in sera of immunized mice were determined by indirect enzyme-linked immunosorbent assay (iELISA). Briefly, U bottom 96-well microtiter plates were coated with 100 µl of 10^6^ TCID_50_ inactivated EV71-Fuyang viruses in carbonate-coating buffer (15 mM Na_2_CO_3_, 35 mM NaHCO_3_, pH 9.8). The plates were incubated at 4°C overnight followed by incubation with 1% BSA in PBS containing 0.05% tween20 (PBS-T) for 1 h at room temperature to prevent non-specific binding. 1000 times diluted sera from immunized mice were then added to plates in duplicates and incubated for 1.5 h at room temperature. After three times washing with PBS-T, horseradish peroxidase (HRP) conjugated goat anti-mouse IgG antibody (1∶2000, DAKO) was added into each well. The reaction was developed by 100 µl TMB substrate (3, 3′, 5, 5′-etramethylbenzidine) for 10 min incubation in a dark room, and then terminated by adding 50 µl 2M H_2_SO_4_. The optical densities at 450 nm were determined with a microplate absorbance reader (Sunrise, Tecan).

### Virus Neutralization Titer (NT) Assay

The presence of neutralizing antibodies against EV71 was assayed by a microneutralization test *in vitro*. First, the mouse sera were incubated at 56°C for 30 min to inactivate the complement. 25 µl of serial two-fold dilutions of serum was mixed with 25 µl of 100 TCID_50_ of virus, and incubated at 37°C for 2 h to neutralize infectious viruses. The mixtures were then transferred to 96-well plates with more than 90% confluent monolayers of RD cells in DMEM containing 5% FBS. After incubation for 5 days at 37°C, the neutralizing antibody titer was read as the highest dilution of serum that completely inhibited virus growth. The experiment was done in duplicates and the average neutralization titer was recorded.

## Results

### Generation of infectious EV71 virus from synthetic cDNA using the human RNA polymerase I system

The plasmid pJET-EV71-Fuyang containing the genomic cDNA of EV71 under the high-fidelity hRNA pol1 promoter was successfully generated according to the method described (Figure 1A). After direct transfection into RD cell, the genomic cDNA of EV71 was transcribed into genomic RNA of EV71 and translated into EV71 polypeptides. The polypeptides were then self-cleaved into functional proteins which successively replicated their genomic RNA and assembled into infectious EV71 virus particles. The viral protein VP1 was detected by guinea pig anti-VP1 sera after transfection of pJET-EV71-Fuyang and two serial passages (Figure 1B). It was obvious that the virus could be recovered from RD cells and was infectious due to the increasing amount of VP1 in progressive passages as seen by IFA. The titer of *in vitro* generated EV71-Fuyang increased with serial passages, and reached a maximum of 3×10^8^ TCID_50_/ml (data not shown). The *in vitro* generated EV71 viruses caused similar CPE in RD cells as other wild-type EV71 viruses. Furthermore, the sera collected from mice immunized with the *in vitro* generated EV71-Fuyang neutralized the wild-type EV71 strains from different subgenogroups which demonstrated that the virus retained immunogenic sites. For 3 chimeric EV71 viruses EV71-Fuyang(VP1-B1), EV71-Fuyang(VP1-B3) and EV71-Fuyang(VP1-C3), the same IFA results and virus titers were observed (data not shown).

### Confirmation of gp64-VP1 expression in insect cells

**Figure 2:**
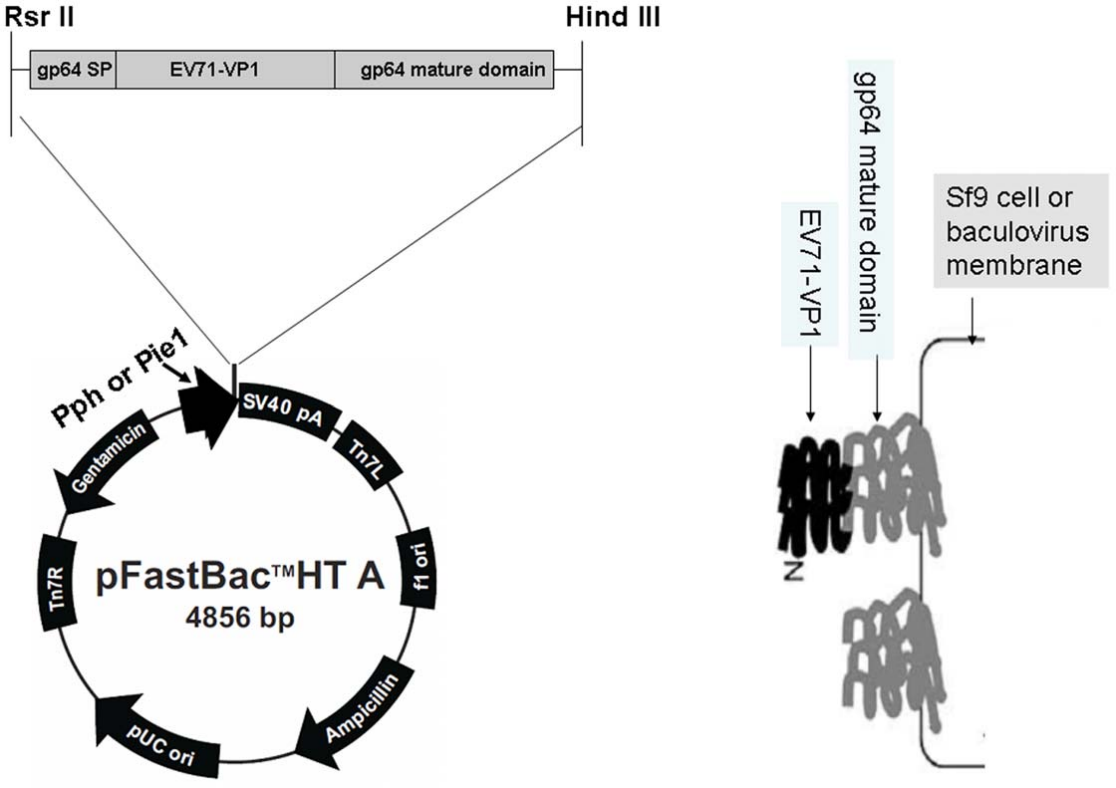
Construction of plasmids for gp64-VP1 expression by baculovirus. The VP1 gene was incorporated between gp64 signal peptide and gp64 mature domain by overlapping PCR. The recombinant gene gp64-VP1 was then inserted into pFastBac HT A. The resultant plasmids were designated pFast-Pph-gp64-VP1. The polyhedrin promoter (Pph) was later replaced by WSSV ie1 promoter which is active in both Sf9 and mammalian cells. Like gp64 protein, the recombinant protein gp64-VP1 should be transported to the surface of the infected Sf9 cells, and incorporated into the surface of recombinant baculoviruses.

The correctness of pFast-Pph-gp64-VP1 and pFast-Pie1-gp64-VP1 was confirmed by DNA sequencing. After transformation into DH10Bac, the recombinant baculoviral genomic DNA Bac-Pph-gp64-VP1 and Bac-Pie1-gp64-VP1 was successfully selected and purified. Sf9 III cells were then transfected and the recombinant baculoviruses expressing gp64-VP1 protein were generated. Our strategy predicted that the gp64 SP would facilitate the transport of the gp64-VP1 to the plasma membrane and be cleaved, thus exposing VP1 on the outer surface of Sf9 cells. Furthermore, the mature domain of gp64 would enable the gp64-VP1 to anchor in the plasma membrane, and then mediate the incorporation of gp64-VP1 into the baculovirus envelope (Figure 2).

**Figure 3:**
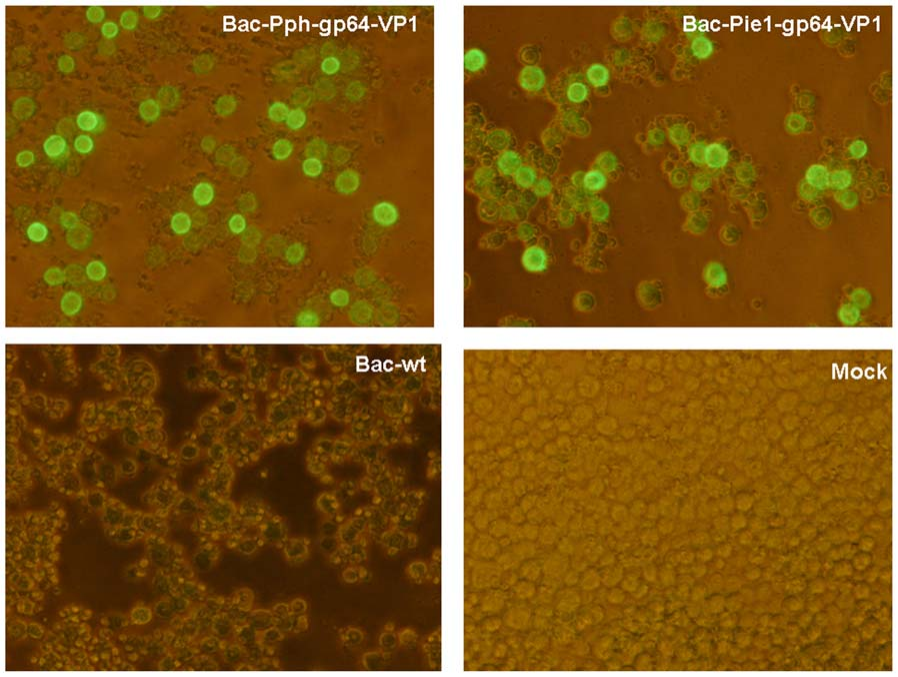
Expression of gp64-VP1 proteins in Sf9 cells. Fresh Sf9 cells were infected by the recombinant baculoviruses Bac-Pph-gp64-VP1 and Bac-Pie1-gp64-VP1 as well as wild-type Baculovirus Bac-wt. Four days post-infection, all infected cells showed distinctive CPE, while mock cells did not. Cells were fixed and incubated with guinea pig anti-VP1 sera followed by FITC-conjugated anti-guinea pig IgG antibodies for IFA. The cells infected by baculovirus Bac-Pph-gp64-VP1 or Bac-Pie1-gp64-VP1 showed positive signals, while no staining was observed in cells infected by wild-type baculovirus or mock-infected cells.

To examine the expression of the fusion protein gp64-VP1 in Sf9 cells, the cells were infected by the recombinant baculoviruses Bac-Pph-gp64-VP1, Bac-Pie1-gp64-VP1 or Bac-wt which were collected from the transfection supernatant. Four days post-infection, the cells were fixed by methanol/acetone and expression of recombinant proteins was detected by guinea pig anti-VP1 sera in IFA. In contrast, no signal was detected by anti-VP1 sera in the negative controls (Bac-wt infected Sf9 cells and mock infected Sf9 cells) (Figure 3).

**Figure 4:**
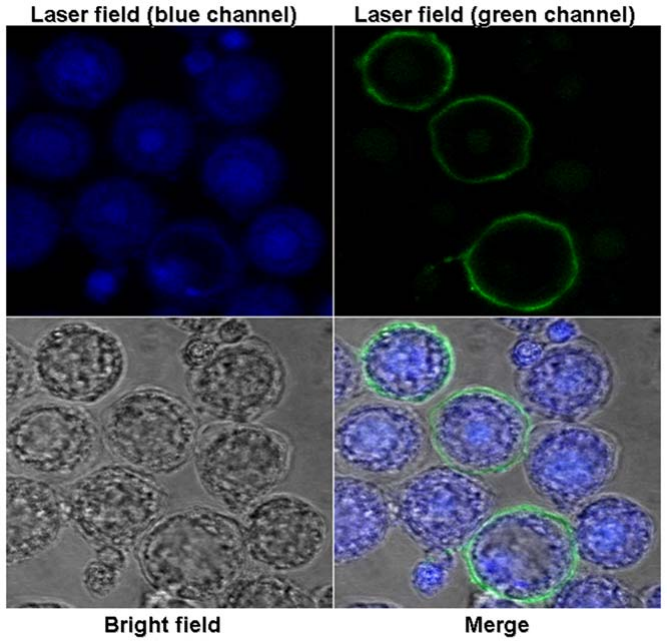
Anchoring of gp64-VP1 on the surface of the infected Sf9 cells. The cells were cultured on sterile glass bottom microwell dishes, infected with baculovirus Bac-Pie1-gp64-VP1 at a MOI of 0.2, and subjected to immunofluorescence staining followed by confocal microscopy 3 days post-infection. The blue channel showed the stained nuclei of Sf 9 cells, whereas the green channel showed the localization of gp64-VP1 fusion protein on the plasma membrane of infected cells. In contrast, the uninfected cells were not detected by anti-VP1 sera.

To determine whether the recombinant gp64-VP1 was properly translocated to the cell surface, the cells were cultured on glass bottom microwell dishes infected with recombinant baculoviruses at an MOI of 0.2, and subjected to IFA at 3 days post-infection. The infected cells were observed under a confocal microscope. As shown in Figure 4, gp64-VP1 was detected in Sf9 cells infected by Bac-Pie1-gp64-VP1 (cells infected by Bac-Pph-gp64-VP1 showed the same results, data not shown), but not in non-infected cells with guinea pig anti-VP1 serum. The gp64-VP1 protein mainly localized within the plasma membrane of Sf9 cells, demonstrating the successful anchoring of gp64-VP1 on the surface of the infected cells.

### Display of gp64-VP1 proteins on the baculovirus envelope

**Figure 5:**
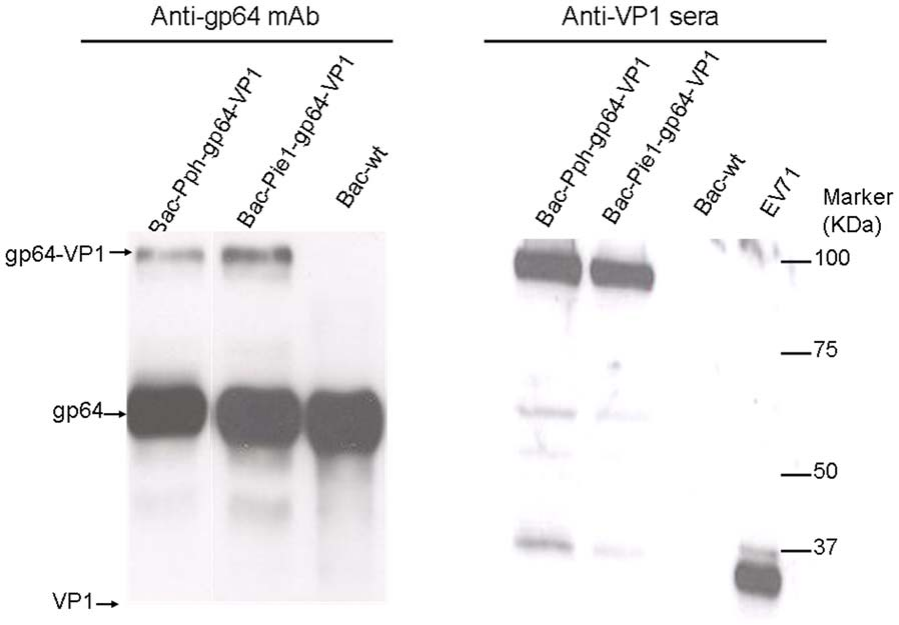
Confirmation of gp64-VP1 expression in purified baculoviruses by Western blotting. Purified baculoviruses Bac-Pph-gp64-VP1, Bac-Pie1-gp64-VP1, Bac-wt and inactivated EV71 were separated on 8% SDS-PAGE gel and transferred onto nitrocellulose membranes. Anti-gp64 monoclonal antibodies (mAbs) and guinea pig anti-VP1 sera were used to detect gp64 and VP1 antigen, respectively.

**Figure 6:**
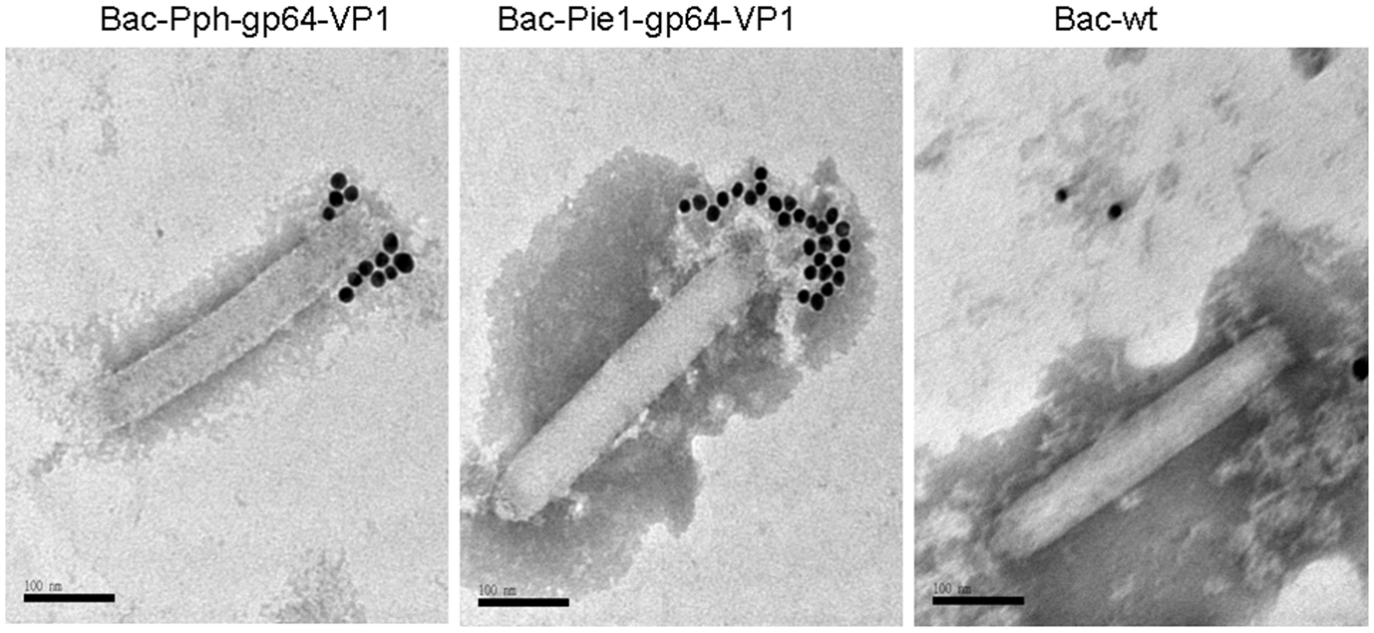
Immunogold electron micrographs of purified recombinant baculoviruses. gp64-VP1 was detected by guinea pig anti-VP1 sera as the primary antibody and anti-guinea pig IgG conjugated with 10 nm gold particles as the secondary antibody (magnification at 200,000x). Gold particles attached to the surface of heads of recombinant baculoviruses Bac-Pie1-gp64-VP1 and Bac-Pph-gp64-VP1 expressing gp64-VP1. In contrast, no gold particles were detected on the surface of wild-type baculoviruses Bac-wt.

To confirm whether the gp64-VP1 was successfully displayed on the baculovirus envelope, recombinant baculoviruses Bac-Pph-gp64-VP1 and Bac-Pie1-gp64-VP1 were purified by two rounds of sucrose gradient ultracentrifugation. Finally, the recombinant baculoviruses were resuspended in PBS at a concentration of 10^8^ pfu/100 µl. Incorporation of recombinant proteins into the baculovirus particles was analyzed by Western blotting. The gp64-VP1 fusion protein was detected by both anti-gp64 mAb and anti-VP1, at a molecular weight of about 98 kDa (Figure 5). Furthermore, the recombinant gp64-VP1 on the baculovirus envelope was visualized by immunogold electron microscopy using primary anti-VP1 sera and secondary antibodies conjugated to 10 nm gold particles. In Figure 6, gold particles on the surface of baculoviruses Bac-Pph-gp64-VP1 and Bac-Pie1-gp64-VP1 indicated the incorporation of gp64-VP1 into the recombinant baculoviral envelop. In contrast, no gold particles were observed on the surface of wild type baculovirus Bac-wt.

### EV71-specific antibodies levels in sera of immunized mice

**Figure 7:**
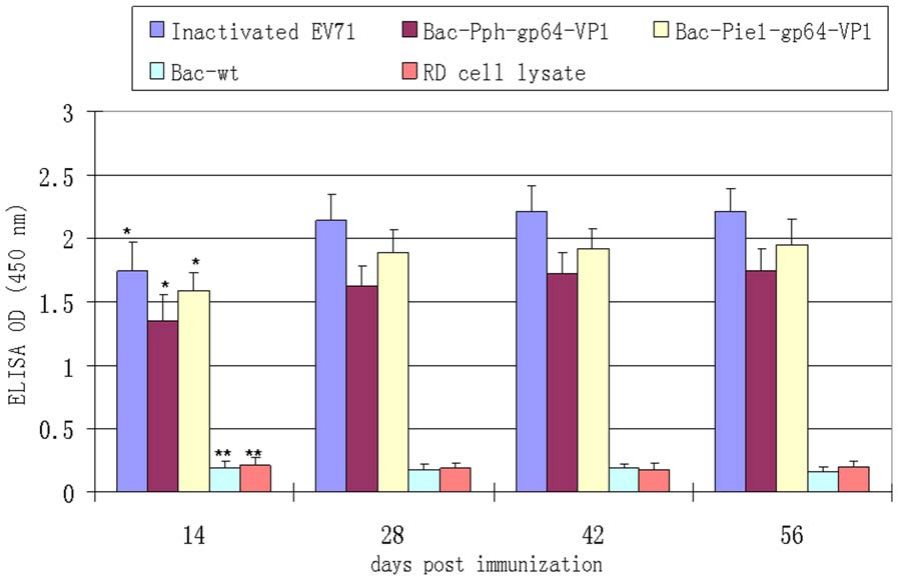
Measurement of EV71-specific IgG antibody levels by indirect ELISA. Five groups of mice, each including 6 female BALB/c mice, were immunized subcutaneously twice at days 0 and 21 with 100 µl solution containing 20 µg of inactivated EV71, 10^8^ pfu of purified Bac-Pph-gp64-VP1, 10^8^ pfu of purified Bac-Pie1-gp64-VP1, 10^8^ pfu of purified wild-type baculovirus Bac-wt and RD cell lysate, respectively, mixed with 100 µl adjuvant. Each column represents the arithmetic mean value (n6) ±SD (*p<0.001; **p<0.01).

We proceeded to explore whether the *in vitro* generated EV71-Fuyang and the gp64-VP1 displayed on the baculovirus envelope could serve as immunogens *in vivo*. Total IgG antibody levels in antisera were assayed by indirect ELISA by coating whole inactivated EV71 virus onto U bottom 96-well plates. The antisera from mice immunized with Bac-wt, or RD cell lysate were used as negative controls. The mice immunized with purified Bac-Pph-gp64-VP1 and Bac-Pie1-gp64-VP1 generated an adequate antibody response against EV71. Meanwhile, the inactivated EV71-Fuyang elicited the highest EV71 antibody levels. In contrast, injection with Bac-wt or RD cell lysate did not elicit specific antibodies against EV71 as O.D. values were below 0.2 in ELISA (Figure 7). The level of specific antibodies against EV71 reached a maximum at 28 days post-immunization and remained at a high level until 56 days post-immunization. The EV71 antibody level elicited by baculovirus Bac-Pie1-gp64-VP1 was slightly higher than that of Bac-Pph-gp64-VP1.

### Neutralization titers of sera of immunized mice

**Figure 8:**
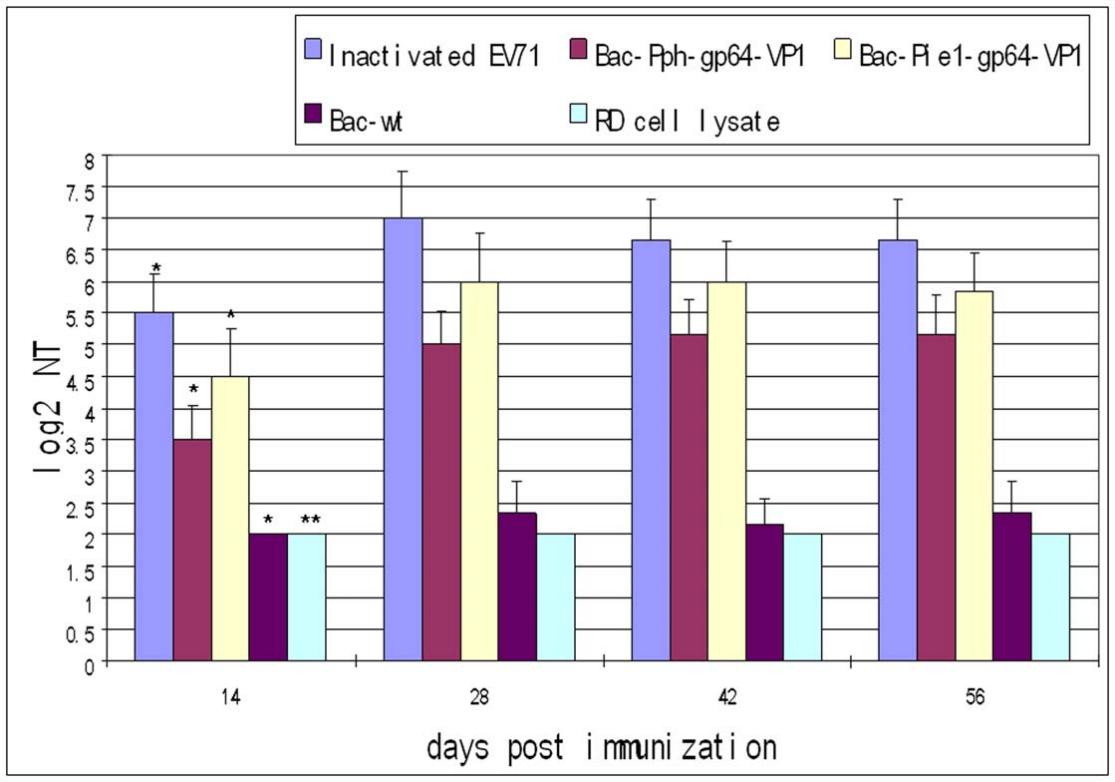
Neutralization titers of the sera of immunized mice. Inactivated EV71 virus, purified baculoviruses Bac-Pph-gp64-VP1 and Bac-Pie1-gp64-VP1 were used in immunization. Wild-type baculovirus (Bac-wt) and RD cell lysate were used as negative controls. Each column represents the arithmetic mean value (n6) ±SD (*p<0.001; **p<0.05). For inactivated EV71, the neutralization titer against EV71 replication in RD cells readed 1∶128, while our baculovirus surface displayed VP1 elicited neutralization titers of 1∶32 to 1∶64.

The antisera were analyzed in an *in vitro* microneutralization assay to determine their ability to neutralize live EV71-Fuyang replication in RD cells. Consistent with the negligible IgG titer, the neutralization titers induced by the Bac-wt and RD cell lysate antisera were barely detectable (<1∶8). In a time-scale study (Figure 8), the inactivated EV71-Fuyang, Bac-Pph-gp64-VP1, and Bac-Pie1-gp64-VP1 antisera resulted in a neutralization titer profile similar to the antibody titer profile, with neutralization titers culminating at 28 days and persisting for at least 56 days. After two immunizations with adjuvant, the inactivated EV71 virus induced a neutralization titer of 1∶128 against EV71 replication in RD cells, while the baculovirus Bac-Pie1-gp64-VP1 and Bac-Pph-gp64-VP1 induced neutralization titer of 1∶64 and 1∶32, respectively.

**Figure 9:**
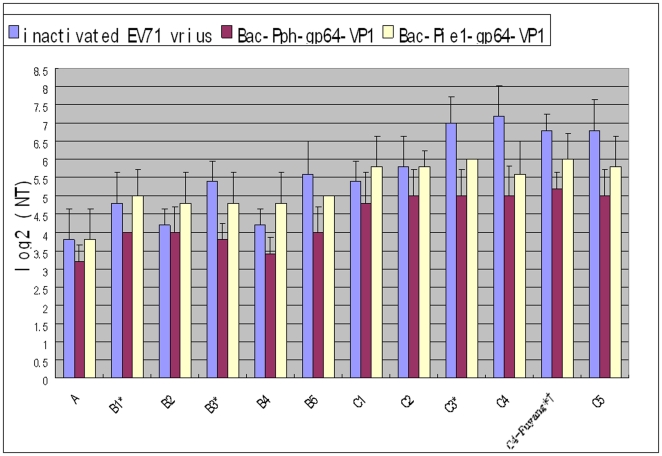
Cross-Neutralization antibodies titers elicited by inactivated virus, Bac-Pph-gp64-VP1 and Bac-pie1-gp64-VP1 in mice at 56 post immunization days against all 11 EV71 subgenogroup strains. Each column represents the arithmetic mean value (n6) ±SD (p<0.001). *generated viruses from synthetic cDNA; †homogenous strain.

These antisera against homologous VP1 of the Fuyang strain exhibited cross-neutralization activities against wild-type EV71 strains from subgenogroups A, B2, B4, B5, C1, C2, C4 and C5 (Table 2). The data showed that Bac-Pie1-gp64-VP1 induced higher neutralization antibody titers than Bac-Pph-gp64-VP1 and had a greater potential to be effective against heterologus EV71 subgenogroups. The neutralization titer of anti Bac-Pie1-gp64-VP1 serum was around 1∶64 against EV71 genogroup C strains and 1∶32 against genogroup B EV71 strains (Figure 9). Therefore, baculovirus-surface displayed VP1 successfully retained antigenic sites of EV71, could elicit good neutralizing antibody response and may serve as a candidate vaccine for EV71.

**Table 2:** List of the EV71 strains used in this study.

Virus strain	Genotype	Origin	GenBank Ref.
BrCr	A	USA	U22521.1
EV71-Fuyang(VP1-B1)*	B1	USA	AF135901
7432/MS/87	B2	USA	U22522.1
EV71-Fuyang(VP1-B3)*	B3	Australia	AF376093
8565/SIN/000009	B4	Singapore	AF316321
NUH0083/SIN/08	B5	Singapore	FJ461781
Y90-3761	C1	Japan	AB433864
1585-Yamagata-01	C2	Japan	AB177812
EV71-Fuyang(VP1-C3)*	C3	South Korea	AY125973
75-Yamagata-03	C4	Japan	AB177813
EV71-Fuynag/02/2008*	C4	China	EU703813
3437/SIN/06	C5	Singapore	GU222654

*Viruses were generated from its synthetic cDNA using human RNA polymerase I system.

## Discussion

In recent years, reverse-genetics systems for the generation of RNA viruses from their cDNA have proven to be of great value for virus research and vaccine development [33], [35], [36], [39]. A previous study has described EV71 recovery methods using infectious RNA which was transcribed *in vitro* from full length cDNA using T7 RNA polymerase [39]. The drawbacks to this method include their complexity, expense, and laboriousness. Additionally, any mutations introduced by proof-reading deficient T7 RNA polymerases used to generate viruses may lead to less viable infectious particles. In this study, we describe an accurate *in vitro* generation of EV71-Fuyang strain in cell culture from its synthetic cDNA by utilizing a eukaryotic cellular RNA polymerase: hRNA pol1. After direct transfection into RD cells, the EV71 cDNA under the hRNA pol1 promoter was transcribed into EV71 genomic RNA by hRNA pol1 inside the cells. This method abolishes the need for troublesome *in vitro* RNA transcription and will be useful for EV71 vaccine research.

Baculovirus surface display is extensively used for vaccine production [40], [41]. In previous studies, we have demonstrated the use of baculovirus surface display technology for candidate vaccine development against highly pathogenic avian influenza [31], [42], [43], and White spot syndrome virus [29]. In the present study, we have shown that baculovirus displaying gp64-VP1 is a candidate vaccine against EV71. A recombinant baculovirus with the immediate-early promoter 1 of WSSV (Bac-Pie1-gp64-VP1) or conventional polyhedrin promoter (Bac-Pph-gp64-VP1) was constructed. Importantly, WSSV ie1 promoter facilitates high-level expression of target protein in insect cells [44]. The nature of ie1 as an immediate-early promoter supports protein expression at the early phase of the baculoviral life cycle, resulting in an enhanced display of the target protein on the baculovirus envelope and it also facilitates efficient transduction of mammalian cells [44], [31]. For the purpose of displaying foreign proteins on the surface of baculovirus particles, gp64 serves as a fusion partner that gets incorporated into the cell membrane and into budded virions together with a chosen target protein [40]. Our results with confocal microscopy revealed that VP1 was able to localize to the plasma membrane of insect cells infected with recombinant baculovirus. In addition, we demonstrated with transmission electron microscopy that baculovirus successfully acquired VP1 from the insect cell membrane via the budding process.

We presumed that VP1 displayed on the baculovirus surface should have been presented in its native form. Hence, we attempted to use Bac-Pie1-gp64-VP1 as a vaccine candidate against EV71 infection. The Bac-Pie1-gp64-VP1 was able to induce systemic immune responses in the vaccinated mice, as indicated by the high level of VP1-specific IgG antibodies in ELISA. *In vitro* microneutrolization assay revealed that Bac-Pie1-gp64-VP1 induced a neutralization titre of 1∶64 against homologous EV71 C4 strain, which is comparable to the neutralization titre induced by inactivated EV71. The EV71 neutralizing antibody level elicited by baculovirus Bac-Pie1-gp64-VP1 was slightly higher than that of Bac-Pph-gp64-VP1. This may be due to the increased activity of WSSV ie1 promoter inside the mouse cells leading to the expression of extra gp64-VP1. In our previous study, we have reported that baculovirus displaying influenza hemagglutinin under the control of ie1 promoter can efficiently transduce intestinal epithelial cells *in vivo* [31]. However, a more detailed study is required to address the role of different promoters in the efficacy of baculovirus surface displayed vaccine. One important issue is whether VP1 subunit vaccines directed against one EV71 genetic subgroup will provide cross-protection against all others since available data are contradictory [45], [46]. The circulating EV71 subgenogroups are changing rapidly, and knowing whether immunity to one subgenogroup confers protection against others is crucial, as it is preferable to have a vaccine that can broadly protect from different subgenogroups of EV71. We demonstrated that antibodies induced by the Bac-Pie1-gp64-VP1 vaccine of the C4 subgenogroup can neutralize other heterologous subgenogroups with 2 to 4 fold difference. This finding agrees with those of other studies showing that hyperimmune animal sera as well as human patient sera derived from one particular subgenogroup exhibit broad neutralizing activity against other subgenogroups [46]–[49]. These observations show the broad efficacy of the Bac-Pie1-gp64-VP1 vaccine against EV71. One limitation in EV71 vaccine development is the lack of a good mouse model of human disease. Adult mice are resistant to infection. Although suckling mice are susceptible, by the time immunity develops after inoculation, the animals have matured and become resistant to infection. Owing to unavailability of a suitable animal model at our facility, viral challenge experiments were not performed. However, the high neutralization titers of our antisera in vitro indicates that they should offer passive protection to suckling mice. Based on the literature of EV71 vaccine studies, antisera with neutralization titers of 1∶64 were able to protect suckling mice in a passive protection study [18], [20]. As the neutralization titers of our antisera are in the same range with those previously described, we would expect an equally good passive protection.

No treatment against EV71 exists, but by analogy with poliomyelitis, vaccination probably offers the best option for EV71 control. Various types of vaccines are being investigated, including inactivated whole-virus, live attenuated, virus-like particle (VLP), and DNA vaccines. All vaccine prototypes are in the early stages of development, with the most advanced undergoing preclinical trials in mice and non-human primates [50]. These candidates include an avirulent EV71 vaccine [51], an EV71-like particle vaccine [52], a DNA vaccine [17], [22] and a recombinant VP1 protein vaccine derived from milk of engineered transgenic mice to produce capsid proteinVP1 [19]. All these vaccines elicited good immune responses in vaccinated mice. However, overexpression of these proteins has been associated with poor solubility, improper folding and the need of expensive purification methods, while live attenuated EV71 vaccine harbors a potential risk of reversion. As reported in candidate EV71 vaccine strain EV71 (S1-3), the vaccine strain itself caused mild neurological symptoms (tremor) in cynomolgus monkeys and entered the spinal cord, which indicated that further work on attenuation is needed [53]. Among the various vaccine candidates, inactivated whole virus vaccines are in some ways most ready to develop further, because the principles of vaccines based on inactive whole virus are well-established. However, inactivation usually destroys epitopes and reduces the efficiency of a vaccine. Furthermore, many bio-safety concerns remain unsolved, such as cases of paralytic polio caused by polio vaccination [54]. An EV71 vaccine can widely be used across the whole of Asia if it is affordable, easily produced, and readily available. Importantly, Bac-Pie1-gp64-VP1 vaccine is efficacious in inducing high neutralization titres, subgenogroup cross-neutralization, and does not require either sophisticated biocontainment infrastructure or downstream purification process for mass production. In addition, the VP1 of any new subgenogroup could be converted into an efficient vaccine with this technology in a short period of time. Considering the above facts, we conclude that Bac-Pie1-gp64-VP1 vaccine could be an ideal choice to reduce EV71-associated morbidity and mortality in the Asia-Pacific region and beyond.
